# Anatomy of a bioengineered human pigmented skin equivalent to provide fundamental insights into skin tone melanin dynamics

**DOI:** 10.1111/joa.70026

**Published:** 2025-07-23

**Authors:** Paola De Los Santos Gomez, Ranjit Rai, Pamela Ritchie, Lucy Smith, Amy Simpson, Kirsty Goncalves, Stefan Przyborski

**Affiliations:** ^1^ Department of Biosciences Durham University Durham UK; ^2^ Reprocell Europe Ltd. Glasgow UK

**Keywords:** 3D cell culture, human skin equivalents, melanin organisation, melanin transfer, melanogenesis, microanatomy, skin pigmentation

## Abstract

Full‐thickness skin equivalents provide a platform for preclinical screening, streamlining the clinical trial process and reducing the need for animal testing while also providing a tool capable of fundamental insights into skin biology. Understanding the specific role of melanin dynamics across various skin tones is essential not only to better understand its function in photoprotection but is also better representative of a diverse population. Although pigmented skin equivalents (PSEs) have been reported in the literature, they rarely recapitulate the structural location of melanin within native keratinocytes, which is pivotal to its photoprotective role. This is due in part to the reliance of existing technologies on exogenous or animal‐derived extracellular matrix (ECM) constituents or the complete lack of a dermal compartment. In this study, we describe the development of novel PSEs representative of skin pigmentation phenotypes *in vitro,* which comprise fibroblast‐secreted endogenous ECM and a differentiated, well‐organised epidermis that resembles diverse skin tones. We demonstrate that these skin tones display morphological differences at a gross, histological and ultrastructural level. We then utilised the system to provide fundamental insights into the processes of melanogenesis, melanin transfer from melanocytes to keratinocytes, supranuclear cap formation and melanosome organisation within the epidermis. Quantification of melanosome dynamics allowed for comparison to native tissue and among skin tones, providing a detailed comparison among experimental conditions. This innovative technology enables a wide range of applications, such as studying pigmentation mechanisms in skin responses to external stimuli, disease modelling and drug testing involving the interactions between the epidermis and dermis.

## Graphical abstract

Human pigmented skin equivalents can be created in vitro to resemble alternative skin tones. Such models can be used to study skin pigmentation processes, structural changes and defined patterns among different skin tones, which correlate with the diversity of pigmentation in native skin.

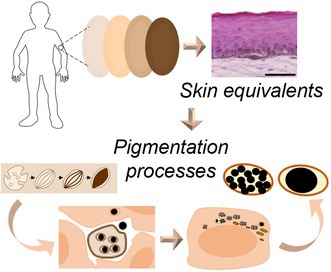

## 1 INTRODUCTION

In recent years, there have been significant advances in the development of in vitro skin equivalents that recapitulate different aspects of human skin anatomy and physiology. Human skin equivalents represent a valuable tool for biomedical and commercial applications, including the study of fundamental biology, mechanisms implicated in skin ageing (Weinmüllner et al., 2020), skin pigmentation (Duval et al., 2014), wound healing (Hofmann, Fink, et al., 2023), skin microbiota crosstalk (Rademacher et al., 2018), disease mechanisms (Brauchle et al., 2013; Cario‐André et al., 2006; Jang et al., 2023; Rioux et al., 2021; Salducci et al., 2014; Sriram et al., 2018), exposure to exogenous stressors (Bechetoille et al., 2007; Bernerd et al., 2012), skin care (Lejeune et al., 2008; Thompson et al., 2022) and drug testing (Bourland et al., 2018; Hardwick et al., 2020).

Full‐thickness skin equivalents predominantly containing keratinocytes and fibroblasts have been increasingly reported in the literature (Hofmann, Schwarz, et al., 2023). However, they do not accurately reflect the complex anatomical and physiological interactions between multiple supporting cell types present in the native tissue. Therefore, more complex skin equivalents have been established through incorporation of specialised skin cells such as immune cells, melanocytes, endothelial cells, neurons, adipocytes and dermal papilla cells to better replicate the cellular interactions integral to human skin tissue functionality (Abaci et al., 2018; Freer et al., 2023; Goncalves et al., 2023; Guo et al., 2022; Kim et al., 2019; Kosten et al., 2015; Mori et al., 2017). Although the integration of melanocytes into human skin equivalents has become increasingly popular (Bernerd et al., 2012; Cario‐André et al., 2000, 2007; Choi et al., 2010; Duval et al., 2001; Duval et al., 2003, 2012; Michalczyk et al., 2018; Yamaguchi et al., 2006, 2008), many limitations are associated with current approaches, including reliance on basic collagen hydrogels or de‐cellularised dermises as matrices, which are often inconsistent with the dermis in vivo. This is particularly problematic as dermal–epidermal crosstalk, basement membrane formation and biochemical interactions with the ECM do not correctly form, which are paramount for the correct production and distribution of melanin within the epidermis (Cario‐André et al., 2006; Duval et al., 2014; El Ghalbzouri et al., 2005, 2009; Goncalves et al., 2023; Hedley et al., 2002; Lee et al., 2003).

The primary biological role of melanin within the epidermis is to provide photoprotection through accumulation in the perinuclear area as supranuclear caps (SNCs), capable of protecting keratinocyte DNA from ultraviolet radiation (UVR)‐induced damage (Cario‐André et al., 2000; Kobayashi et al., 1998). Additionally, the type, quantity and distribution of melanin through the tissue are factors responsible for facultative pigmentation, which can be influenced by external stimuli and are responsible for the appearance of skin tone (Tadokoro et al., 2003, 2005; Yamaguchi et al., 2006). Melanin is produced by melanocytes and transferred to neighbouring keratinocytes, where SNCs are formed. However, the specific mechanism of melanin transfer is still disputed, with endo/exocytosis and phagocytosis among the proposed mechanisms (Moreiras et al., 2020, 2022; Tarafder et al., 2014). Although the fundamental dynamics of melanin production and distribution throughout the tissue are widely accepted, specific underpinning cellular and molecular mechanisms remain poorly understood.

Understanding the kinetics associated with the production, transfer and distribution of melanin is essential to better understand its photoprotective properties, as its location within keratinocytes and role in SNCs are essential for its function (Kobayashi et al., 1998). The location of melanin in the epidermis is also known to differ between skin tones, with darker skin tones containing larger melanin deposits extending into the upper epidermal layers (Del Bino et al., 2015). In contrast, lighter skin tones contain more punctate melanin deposits, more frequently distributed in the epidermal basal and spinous layers. This location and distribution of melanin are consistent with the propensity of skin tones to burn and their susceptibility to UVR‐induced damage (Del Bino et al., 2006), highlighting the importance of better understanding the biological processes that govern melanin dynamics.

We have previously reported the development of a PSE constructed upon a dermal foundation of human fibroblasts that neo‐synthesise a complex network of ECM proteins and form an anatomically correct basement membrane de novo (Roger et al., 2019). This more accurately recapitulates the architecture and function of pigmented human tissue in vitro, facilitating the formation of SNCs, UVR protection and response to hyper‐ and hypo‐pigmentation stimuli (De Los Santos Gomez et al., 2024; Goncalves et al., 2023). In this study, we now utilise this platform as an innovative tool that can provide valuable insights into melanin dynamics and melanosome biology among different skin tones. We integrate melanocytes from donors representative of a variety of skin tones into the epidermis of this established PSE to recapitulate their constitutive pigmentation and to investigate microanatomical differences in the underlying biological processes associated with pigmentation, such as melanosome formation, melanogenesis, melanin transfer, distribution and organisation. Our results highlight the morphological differences between alternative skin tones when skin keratinocytes and fibroblasts are incorporated within a novel three‐dimensional tissue construct, overcoming the limitations of conventional cell culture approaches. They demonstrate that PSEs exhibit close similarities to the microanatomy of in vivo human pigmented skin and emphasise the robust nature of human skin equivalents that offer more personalised strategies to study human skin pigmentation diversity in a controlled manner in vitro.

## 2 METHODS

### 2.1 Cell culture

Human neonatal dermal fibroblasts #1366356 (HDFn, Thermo Fisher Scientific, Loughborough, UK) were maintained in Dulbecco's Modified Eagle Medium (DMEM, Thermo Fisher Scientific) supplemented with fetal bovine serum (FBS, Thermo Fisher Scientific) and L‐glutamine 200 mM (Thermo Fisher Scientific).

Human neonatal epidermal keratinocytes #2018512 and #2286109 (HEKn, Thermo Fisher Scientific) were maintained in EpiLife medium (Thermo Fisher Scientific), supplemented with human keratinocyte growth supplement (HKGS, Thermo Fisher Scientific). HDFn and HEKn were derived from lightly pigmented Caucasian male donors.

Darkly‐pigmented human neonatal epidermal melanocytes #2077650 (HEMn‐DP, ThermoFisher Scientific), moderately‐pigmented human neonatal epidermal melanocytes #2302111 (HEMn‐MP, ThermoFisher Scientific) and lightly‐pigmented human neonatal epidermal melanocytes #2291033 (HEMn‐LP, ThermoFisher Scientific) were maintained in Medium 254® (Thermo Fisher Scientific), supplemented with human melanocyte growth supplement (HMGS, Thermo Fisher Scientific). HEMn were derived from male donors.

Cell lines were maintained at 37°C in a 5% CO_2_ and 95% humidified incubator following the supplier's instructions.

### 2.2 Skin equivalent generation

Human full‐thickness skin equivalents were generated as previously described (De Los Santos Gomez et al., 2024; Goncalves et al., 2023). After 48 h in submerged culture, the PSEs were raised to the air‐liquid interface (ALI) to promote keratinocyte differentiation and stratification and maintained for a further 21 days.

### 2.3 Individual typological angle & melanin index readings

Individual typological angle (ITA) and melanin index (MI) readings were obtained using a colourimeter, the SkinColorCatch (Delfin Technologies, Surrey, UK). Three measurements were taken per skin to provide an average value from different surface areas.

### 2.4 Paraffin wax embedding

PSEs were fixed in 4% paraformaldehyde (Sigma‐Aldrich, Dorset, UK), gradually dehydrated in ethanol and incubated in Histoclear (Scientific Laboratory Supplies, Nottingham, UK), a 1:1 ratio of Histoclear and paraffin wax (CellPath, Newtown, UK) and 100% molten paraffin wax. Samples were embedded in plastic moulds (Solmedia, Shrewsbury, UK) with paraffin wax. Paraffin wax blocks were sectioned at 5 μm using a rotary manual microtome Leica RM2125RT (Leica Biosystems, Nussloch, Germany). Sections were then mounted onto charged Superfrost Plus microscope slides (Thermo Fisher Scientific).

### 2.5 Histological analysis

Sections mounted on slides were deparaffinised in Histoclear and gradually rehydrated in ethanol.

For Haematoxylin & Eosin (H&E) staining, slides were incubated in Mayer's haematoxylin (Sigma‐Aldrich) for 5 min and rinsed in distilled water for 30 s, followed by an incubation in alkaline ethanol for 30 s. Slides were dehydrated in ethanol to 95% ethanol prior to incubation with eosin (Sigma‐Aldrich) for 30 s. The slides were subsequently dehydrated to 100% ethanol, cleared twice in Histoclear and mounted with Omnimount (Scientific Laboratory Supplies).

Fontana Masson melanin staining was performed using a commercially available kit (Abcam, Cambridge, UK, ab150669) following the manufacturer's instructions.

Histological images were captured using a Leica ICC50 high‐definition camera (Leica Microsystems, Wetzlar, Germany) mounted onto a DM500 Leica microscope (Leica Microsystems). Images were processed using the Fiji software (Schindelin et al., 2012).

Melanin mask was identified in each Fontana Masson‐stained micrograph by segmentation of the epidermis with the Sauvola thresholding method using the Fiji software.

### 2.6 Epidermal whole‐mount staining

Epidermal whole‐mount staining and imaging were achieved using the previously described methodology (De Los Santos Gomez et al., 2024). Images were processed using the Fiji software.

### 2.7 Electron microscopy

For TEM, samples were fixed in 2% paraformaldehyde, 2.5% glutaraldehyde in 0.1 M cacodylate buffer (Karnovsky's Fixative) for 1 h at room temperature. They were washed three times for 5 min in 0.1 M cacodylate buffer (pH 7.6) (Agar Scientific) and post‐fixed in 1% osmium tetroxide (Agar Scientific) for 1 h. Samples were washed three times in 0.1 M cacodylate buffer (pH 7.6) and dehydrated and embedded as previously described (Roger et al., 2019).

Ultra‐thin sections were cut from resin blocks using a diamond knife (Agar Scientific) on a UC7 ultramicrotome (Leica) and transferred to 100‐mesh copper, formvar‐coated grids (Agar Scientific). Sections were post‐stained with 1% uranyl acetate (VWR International) in 70% ethanol, washed in water and then stained with Reynolds' Lead Citrate (VWR International) for visualisation.

Images were captured on a Hitachi H7600 transmission electron microscope (Hitachi High‐Tech, Oxford, UK) equipped with an EMSIS Xarosa 20 Megapixel Bottom‐mount CMOS digital camera (Emsis, Münster, Germany), or JEOL 1400 Plus transmission electron microscope (JEOL Limited, Tokyo, Japan) fitted with an Advanced Microscopy Technologies XR16 digital camera (AMT Imaging Direct, Wobum, MA, USA). Images were processed using the Fiji software.

### 2.8 Morphometric melanosome analysis

Morphometric analysis was performed according to the previously reported methodology in TEM studies (Faitg et al., 2020; Hurbain et al., 2018). Shape descriptors and size measurements of melanosomes were obtained by manually tracing from TEM images using the freehand tool in Fiji. The definition of the distinct compartments was based on their morphology. Surface area is reported in μm^2^ × 10^−2^ and perimeter in μm. Circularity is an index of sphericity with values of 1 indicating perfect spheroids, which was calculated by the formula [4*π*·(surface area/perimeter^2^)]. The Feret's diameter represents the longest distance (nm) between any two points within a given structure.

Biometric measurements of such structures were evaluated on randomly selected cell profiles from three independent experiments per phenotype group (highly, moderately and lightly pigmented skin).

### 2.9 Statistics

GraphPad Prism version 10 software for Windows (GraphPad Software, La Jolla, California, USA) was used to measure statistical significance by conducting a two‐way ANOVA analysis. Statistical differences were noted as *, **, *** or **** corresponding to *p* < 0.05, <0.01, <0.001, <0.0001, respectively.

## 3 RESULTS

### 3.1 Development of pigmented skin equivalents representative of varied skin tones

**FIGURE 1:**
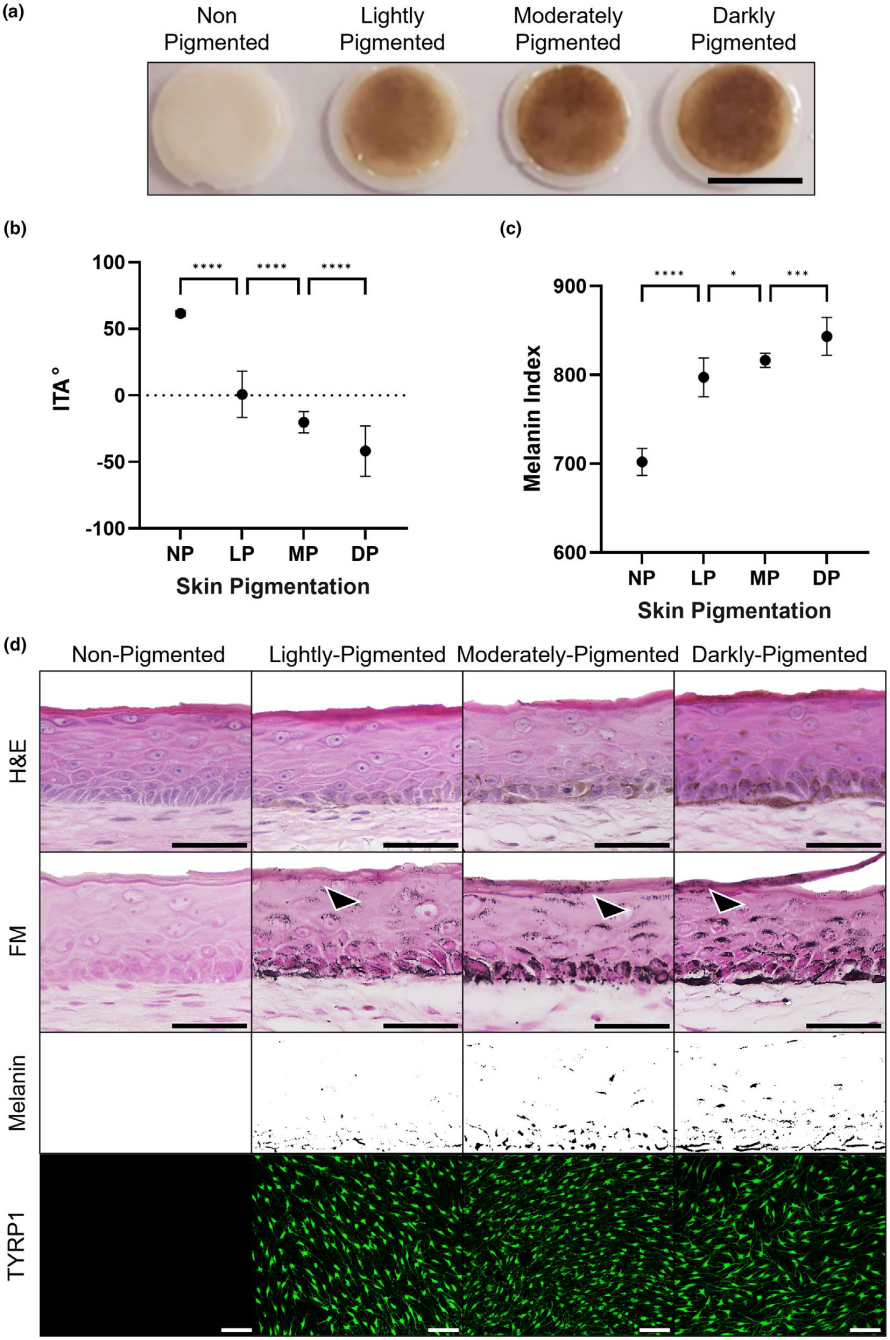
Generation of a skin tone palette in vitro using pigmented human skin equivalents. Representative analysis of pigmented full‐thickness skin equivalents that recapitulate constitutive pigmentation phenotypes. (a) Gross appearance, (b) ITA, (c) MI and (d) histological micrographs demonstrate a significantly darker skin tone and higher levels of melanin, which correlate with increasing pigmentation of the melanocytes incorporated into the construct. Melanin segmentation from Fontana Masson (FM) images (d) demonstrates melanin distribution across epidermal layers in the skin tones. Arrow heads indicate the presence of SNCs. Immunofluorescence micrographs of TYRP1‐stained whole‐mounted epidermises demonstrate even distribution of melanocytes within the basal layer of the pigmented skin equivalents. GraphPad Prism software was used to generate the graphs displaying the mean ± SEM from 2 independent experiments with 3 skin equivalents per skin tone and 3 measurements per skin equivalent. An ordinary 2‐way ANOVA with Tukey correction for multiple comparisons was used to determine statistical significance, *p < 0.05, ***p < 0.001, ****p < 0.0001. Scale bar: 1 cm (a), 50 μm (d, H&E and FM), 100 μm (d, TYRP1).

Human neonatal melanocytes from lightly, moderately and darkly pigmented donors were successfully incorporated into skin equivalents. Representative images of PSE gross appearance resembled different constitutive pigmentations, reflecting the pigmentation of the respective donor skin tone (Figure 1a). For comparison, non‐pigmented skin equivalents were included in all the analyses. Although the LP‐PSEs appear obviously less pigmented than the MP‐PSEs, the difference in pigmentation between MP‐PSEs and DP‐PSEs is more subtle at a gross level. For this reason, we conducted a more objective evaluation of a variety of skin tone parameters through the use of a dermatological colourimeter, as previously described (De Los Santos Gomez et al., 2024; Goncalves et al., 2023). Individual Typology Angle (ITA) measurements demonstrated a darker skin tone when melanocytes incorporated within PSEs were obtained from donors of darker skin tones (Figure 1b). According to the ITA classification (Del Bino et al., 2006; Del Bino & Bernerd, 2013): the non‐pigmented full‐thickness skin equivalent (average ITA = 61.55°) could be categorised as very light skin; the lightly PSE (average ITA = 0.66°) as brown skin, very close to the tan category; the moderately PSE (average ITA = −20.22°) as brown skin; and the darkly PSE (average ITA = −41.83°) would correspond to the dark skin tone. Similarly, the melanin index (MI) readings demonstrated higher melanin content when darker melanocytes were incorporated into the PSEs (Figure 1c). Pigmentation parameters confirmed that the more pigmented the melanocytes, the darker the skin and the higher the melanin content, despite the PSEs being constructed using keratinocytes from the same donor line (Caucasian).

Histological analysis showed a well‐differentiated and stratified epidermis in all conditions (Figure 1d). Fontana Masson (FM) staining confirmed that all types of melanocytes have been correctly incorporated and localised in the *stratum basale* of the PSEs. It is also evident that melanin was synthesised and transferred to keratinocytes which formed typical SNCs (Figure 1d‐FM). Additionally, differences in melanin deposition were observed in suprabasal keratinocytes between pigmentation phenotypes: the darker the skin, the more defined and larger SNCs (arrowheads). Melanin distribution across the epidermal layers differed with the pigmentation level, as seen by melanin segmentation from FM staining (Figure 1d‐Melanin). Darker PSEs displayed larger SNCs and more melanin across all the epidermal layers. Tyrosinase‐related protein 1 (TYRP1) whole‐mount staining confirmed melanocyte localisation and even distribution across the basal layer of the PSEs (Figure 1d).

**FIGURE 2:**
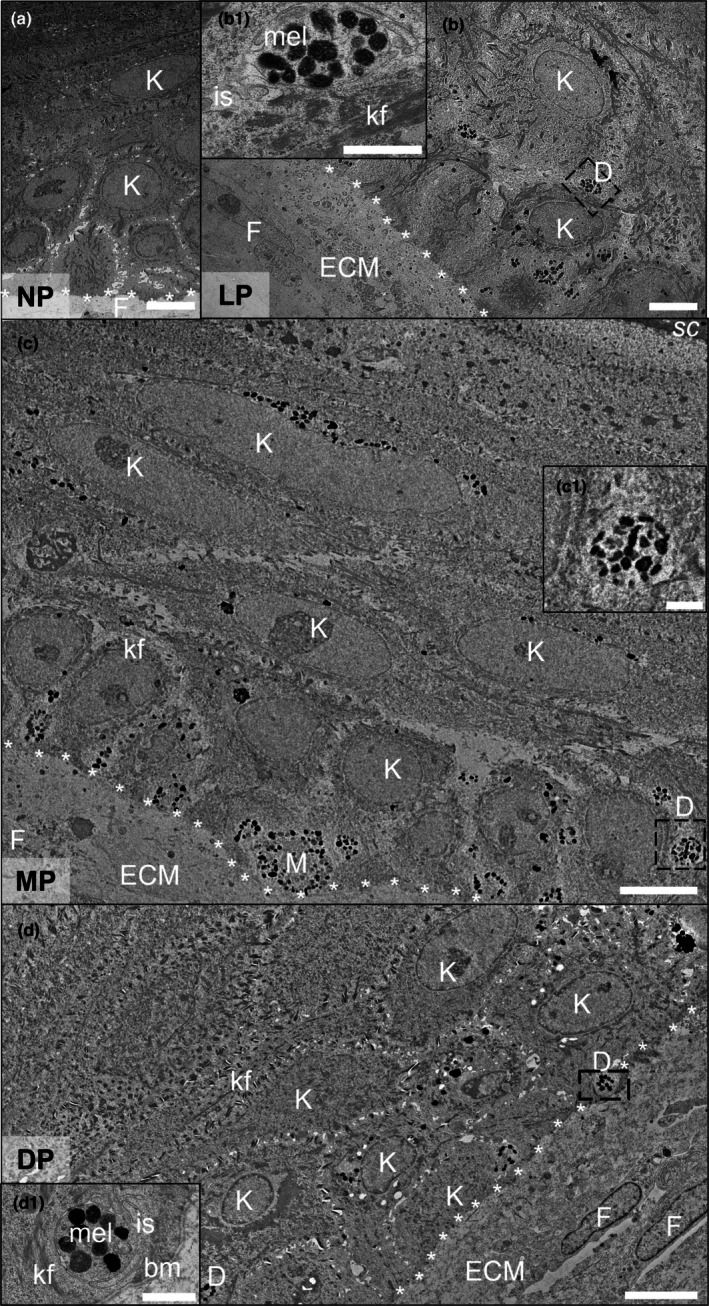
Ultrastructural characterisation of pigmented human skin equivalents. TEM micrographs of sectioned: (a) non‐pigmented (NP) and (b) lightly pigmented (LP), (c) moderately pigmented (MP) and (d) darkly pigmented (DP) skin equivalents. Image data show human fibroblasts (F) surrounded by a network of endogenous extracellular matrix (ECM) within the dermal compartment, and the epidermis containing basal and suprabasal keratinocytes (K) with characteristic keratin filaments (kf). Melanocyte dendrites (D) extend between keratinocytes in pigmented skin equivalents. Asterisks represent the dermal‐epidermal junction. High magnification micrographs of melanocyte dendrites are shown in (b1, c1, d1) containing mature melanosomes (mel). Non‐electron‐dense intercellular space (is) is identified between melanocyte dendrites and keratinocytes. Scale bars: 5 μm (a, b, c, d), 1 μm (b1, c1, d1).

The microanatomy within the dermal and epidermal compartments of the PSEs was assessed by transmission electron microscopy (TEM) (Figure 2). The dermal compartment was identified by fibroblasts surrounded by a large quantity of endogenous ECM. The basement membrane, which forms the dermo‐epidermal junction, was clearly located between both compartments. Keratinocytes from the *stratum basale* and *stratum spinosum* were identified in the epidermal compartment (Figure 2a–d). Profiles of sectioned keratinocytes ranged between 5 to 20 μm in diameter and contained rich amounts of keratin intermediate filaments. Sectioned profiles of melanocyte dendrites extended between keratinocytes and were identified by the presence of melanosomes, electron‐dense aggregates of black particles. This level of ultrastructural analysis of the PSEs showed the correct anatomical location of melanocytes within the epidermis and supporting evidence for their functionality through the production and dendritic transfer of melanin within the bioengineered tissue.

### 3.2 Ultrastructural characterisation of melanosome morphology across alternative skin tones in vitro

**FIGURE 3:**
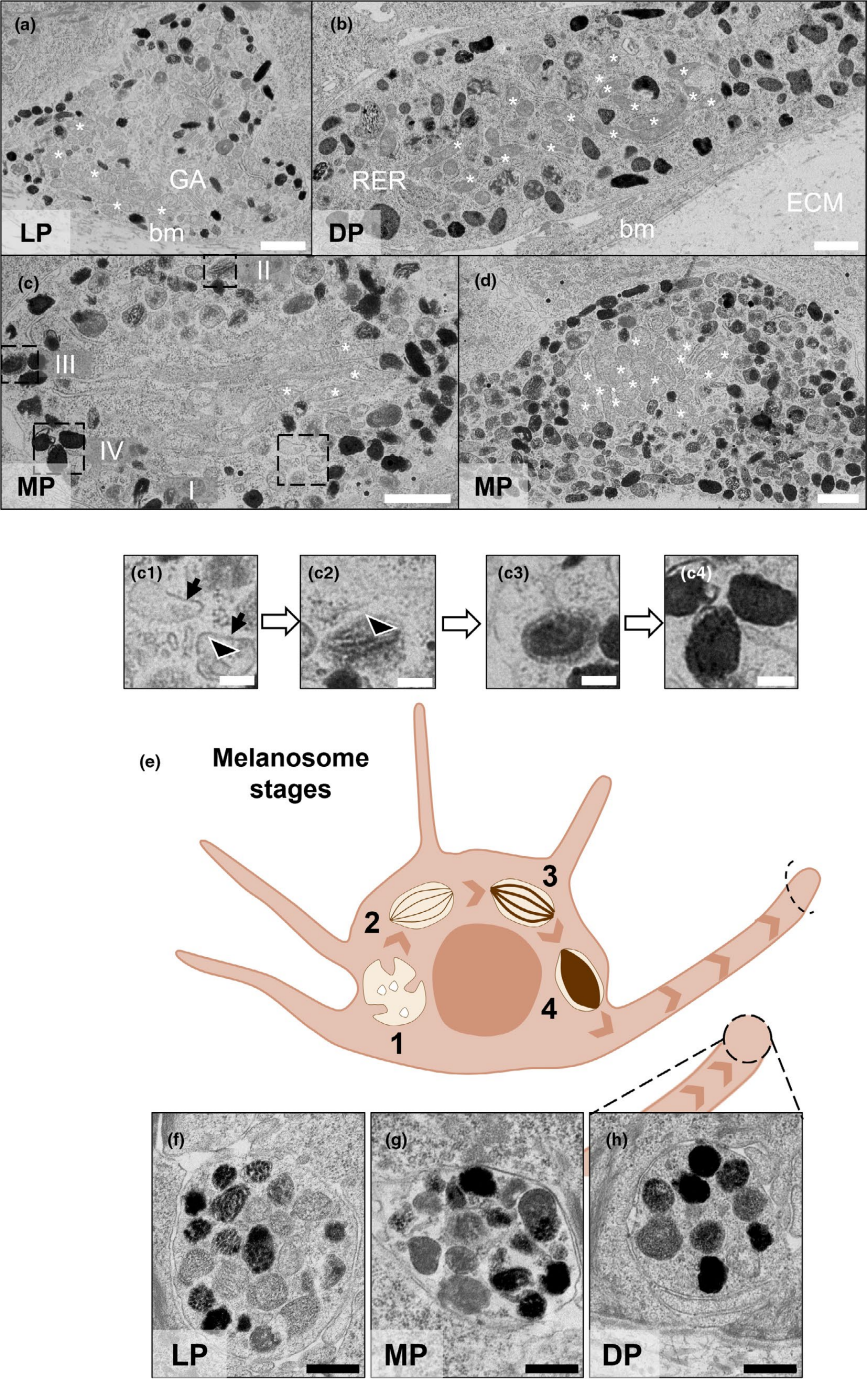
In‐depth analysis of melanocytes found in the pigmented full‐thickness skin equivalents demonstrates melanocyte location at the basement membrane and maturation stages of melanosomes in melanocyte bodies and dendrites. Representative cross‐sectional TEM micrographs of melanocytes in the *stratum basale* of pigmented skin equivalents showing melanosomes at different stages. Asterisks represent mitochondria. (a) Lightly pigmented skin equivalent (LP). (b) Darkly pigmented skin equivalent (DP). (c, d) Moderately pigmented skin equivalent (MP). The boxed regions in (c) show melanosomes at different stages, which are shown at higher magnification in c1–c4. (c1) Stage I melanosomes are identified by a dense bilayered coat (arrows) and intraluminal vesicles (arrowhead). (c2) Stage II melanosomes are identified by the intraluminal proteinaceous fibrils (arrowhead). (c3) Stage III melanosomes show melanin deposition on the fibrils through their thickening and blackening until maturation, which is observed in stage IV (c4). (e) Schematic of melanosome maturation stages and transport to dendrites. Representative cross‐sectional TEM micrographs of melanocyte dendrites of (f) lightly, (g) moderately and (h) highly pigmented skin equivalents. Key: Basement membrane (bm), extracellular matrix (ECM), intercellular space (is), keratinocyte (K), keratin filaments (kf), melanocytes (M), melanosomes (mel), Golgi apparatus (GA), rough endoplasmic reticulum (RER). Scale bars: 1 μm (a, b, c, d), 200 nm (c1, c2, c3, c4), 500 nm (f, g, h).

Different stages of melanosome formation were observed in melanocytes within the PSEs (Figure 3). In Figure 3a–d, cross‐sections of melanocyte bodies were identified by the rich number of melanosomes, lack of keratins and absence of desmosomes. Melanocytes were located in the basal layer of the PSEs, above the basement membrane, consistent with native skin (Figures 2c and 3a–d). Electron‐dense melanosomes at different maturation stages were identified around mitochondria inside the melanocyte cell bodies (Figure 3a–d). Melanosome stages were identified, and examples were shown in Figure 3c1–c4. Stage I melanosomes (Figure 3c1) were round organelles with an electron‐lucent lumen, with few intraluminal vesicles (Le et al., 2021) and a bilayered clathrin coat (Raposo & Marks, 2007) identified as early endosomes. Stage II melanosomes (Figure 3c2) were identified by their parallel or concentric proteinaceous fibrils, which provide an elongated shape and are the site for later‐stage melanin deposition (Hurbain et al., 2008). Stage III melanosomes (Figure 3c3) were identified by the electron‐dense melanin, indicating the initiation of melanogenesis, while stage IV melanosomes include sufficient melanin to mask the proteinaceous fibrils, indicative of melanosome maturation (Figure 3c4). Stages III and IV melanosomes were clearly identified within the dendrites of melanocytes, prior to transfer to neighbouring keratinocytes.

**FIGURE 4:**
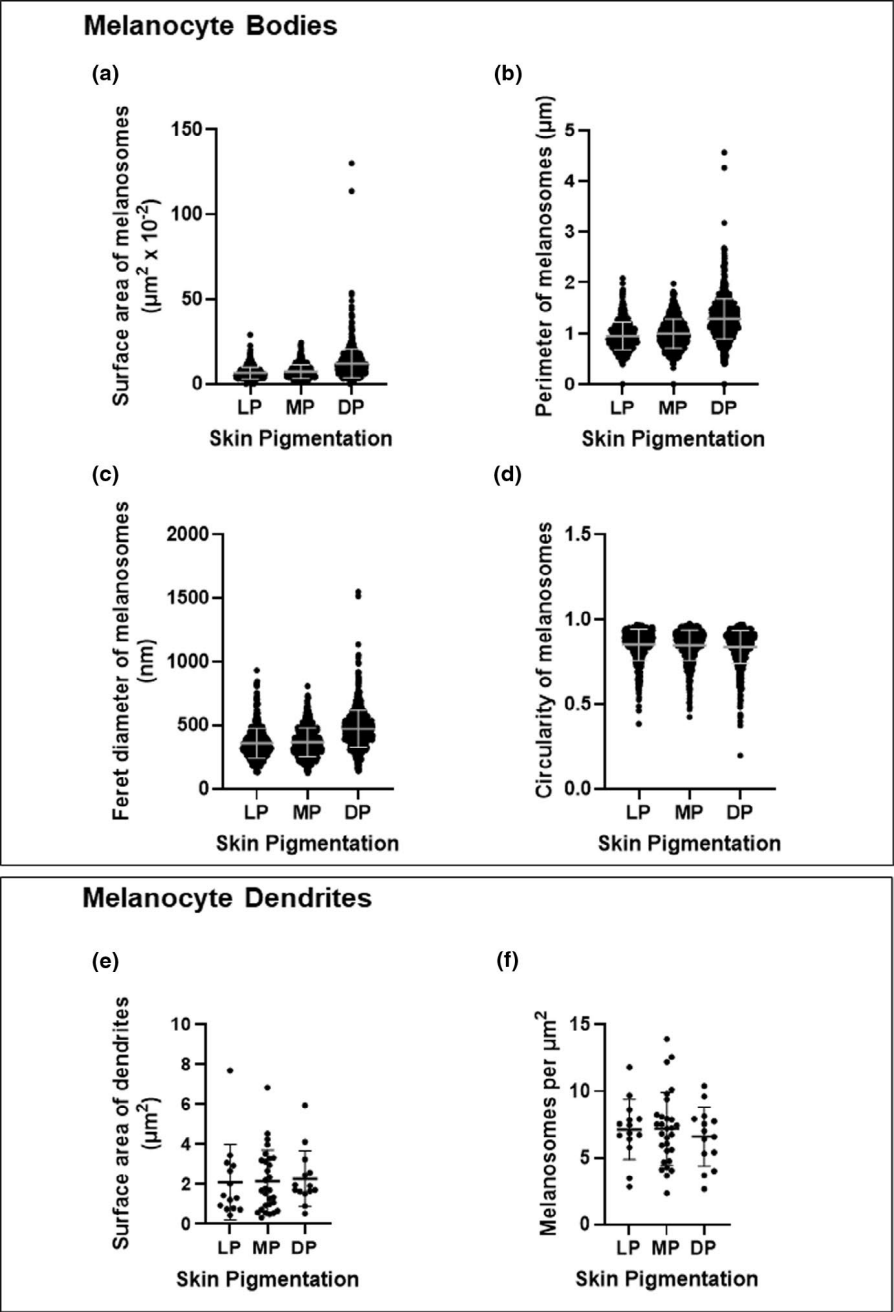
Morphometric analysis of melanosomes in melanocyte cell bodies and dendrites across various skin tone models in vitro. Morphologic analysis of melanosomes in melanocyte body sections in lightly (LP), moderately (MP) and darkly (DP) pigmented skin equivalents: (a) Surface area of melanosomes in melanocyte bodies (μm^2^ × 10^−2^), (b) perimeter of melanosomes in melanocyte bodies (μm), (c) circularity of melanosomes in melanocyte bodies and (d) Feret diameter of melanosomes (nm). LP: *n* = 527 melanosomes, 5 melanocytes; MP: *n* = 664 melanosomes, 5 melanocytes; DP: *n* = 924 melanosomes, 7 melanocytes. Morphologic analysis of melanosomes in melanocyte dendrite sections: (e) Surface area of dendrites (μm^2^) and (f) melanosomes per μm^2^. LP: *n* = 179 melanosomes, 14 dendrites; MP: *n* = 415 melanosomes, 29 dendrites; DP: *n* = 181 melanosomes, 14 dendrites. GraphPad Prism software was used to generate the graphs displaying the mean ± SD.

To investigate whether there is an association between melanosome morphology and skin tone, we measured melanosome area, perimeter, Feret diameter and circularity from images of melanocytes captured on TEM micrographs, as those described in Figure 3a–d. It was expected that melanosome morphology would be smaller and more punctate in lighter skin tones (Minwalla et al., 2001). The surface area average of melanosomes in lightly, moderately and darkly PSEs was measured at 6.41, 7.16 and 11.98 × 10^−2^ μm^2^, respectively (Figure 4a). Similarly, perimeter averages also demonstrated a trend with increased perimeter appearing to correlate with increased pigmentation and were recorded at 0.949, 0.996 and 1.291 μm, respectively (Figure 4b). The Feret diameter represents the most extended length of irregular‐shaped particles such as melanosome cross‐sections. In melanocyte bodies, melanosomes displayed Feret diameter averages of 358.97, 367.31 and 473.23 nm in lightly, moderately and darkly PSEs, respectively (Figure 4c). It was noted that the circularity average of melanosomes showed a modest decrease with skin pigmentation: 0.8516 in lightly, 0.8484 in moderately and 0.8382 in darkly PSEs (Figure 4d). Collectively, these data demonstrated that darker PSEs exhibited the highest average values for surface area, perimeter and Feret diameter and the lower circularity, indicating that the morphology of melanosomes found in melanocyte bodies differs with skin tone.

To investigate differences in melanocyte dendrites among skin tones, quantification was performed on dendrite profiles identified in electron micrographs that represent transverse ultra‐thin sections of the tissue construct, as those illustrated in Figure 3f–h. In order to reduce sampling bias due to the use of transverse sections for this method of analysis, we measured a large number of melanosomes (>150) per classification of tissue construct to strengthen our findings and ensure robust conclusions. Figure 3 shows examples of melanosome sections from dendrites in lightly, moderately and darkly PSEs. Averages of dendrite size ranged from 2.10 μm^2^ in lightly PSEs to 2.27 μm^2^ in darkly PSEs (Figure 4e). Melanosome density per μm^2^ among skin tones comprised averages of 7.13, 7.18 and 6.61 μm^−2^ in lightly, moderately and darkly PSEs, respectively (Figure 4f). These data show a modest trend that dendrite cross‐section sizes generally increase with pigmentation level as melanosome number decreases.

### 3.3 Melanin transfer, distribution and organisation within the pigmented epidermis in vitro

**FIGURE 5:**
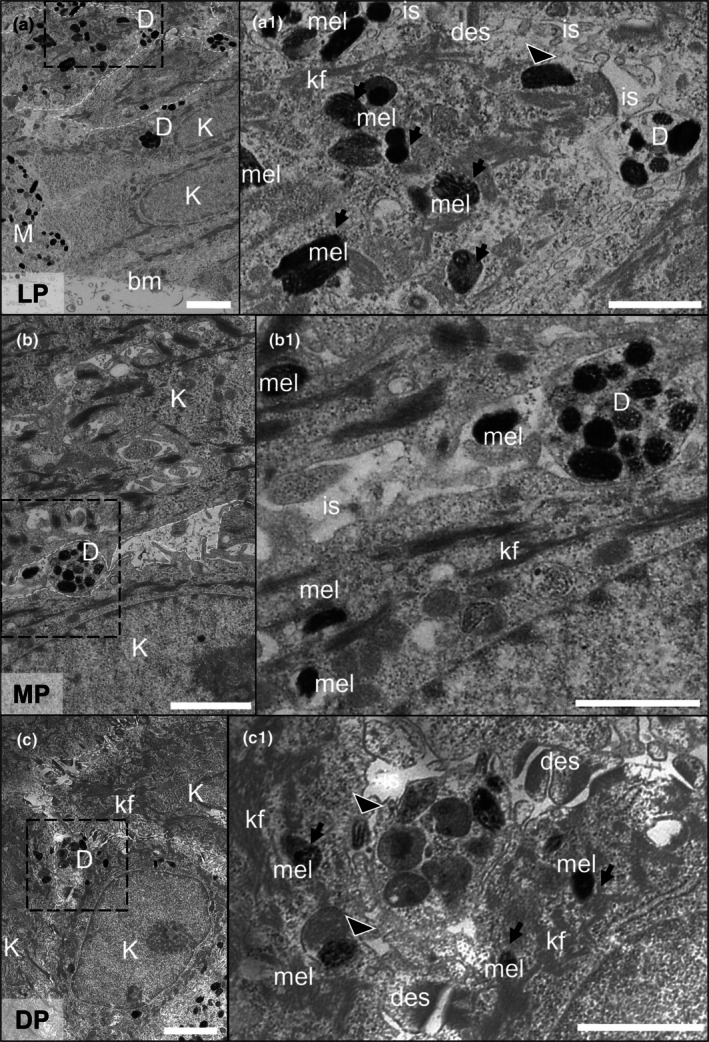
Ultrastructural analysis of melanin transfer through melanocyte dendrites to keratinocytes of human pigmented full‐thickness skin equivalents. Representative TEM micrographs of sectioned human pigmented skin equivalents showing melanocyte dendrites between keratinocytes in (a) lightly (LP), (b) moderately (MP) and (c) darkly (DP) pigmented models. High magnification micrographs of boxed areas in (a), (b) and (c) are shown in (a1), (b1) and (c1), respectively. Images (a1, c1) show melanocores close to dendrites being internalised into keratinocytes (arrowhead) and other melanocores (arrows) identified inside adjacent keratinocytes. Image (b1) shows melanocores (mel) located in the intracellular space between melanocyte dendrite and keratinocytes. Key: basement membrane (bm), dendrite (D), desmosomes (des), intercellular space (is), keratin filaments (kf), keratinocyte (K), melanocore (mel). Scale bars: 2 μm (a, b, c), 1 μm (a1, b1, c1).

Ultrastructural image data repeatedly demonstrated the consistent presence of melanocyte dendrites in close proximity to keratinocytes, providing supporting evidence that the transfer of melanin to keratinocytes from melanocyte dendrites occurs within PSEs *in vitro*. Figure 5 shows examples of micrographs with melanosome‐containing dendrites sandwiched tightly between keratinocytes, in many instances, with directly apposing membranes and minimal extracellular space in between. Once the melanosomes are transferred from melanocytes to the extracellular space, they lose their melanosome membrane and are termed melanocores (Tarafder et al., 2014). Then, they are thought to be internalised by the keratinocyte and are surrounded by a single outer membrane and termed melanokerasomes (Neto et al., 2024). However, this process is disputed, and opposing mechanisms have also been described (Ando et al., 2012; Moreiras et al., 2020, 2022; Singh et al., 2010; Yamamoto & Bhawan, 1994). Figure 5b shows a melanocore situated in the intercellular space between melanocyte and keratinocyte plasma membranes, while Figure 5a,c display some melanocores (arrowheads) having been internalised within keratinocytes. These data support the concept of coupled melanocyte exocytosis and keratinocyte‐mediated melanin phagocytosis as a proposed mechanism of transfer in human skin in vivo (Moreiras et al., 2020, 2022; Tarafder et al., 2014).

**FIGURE 6:**
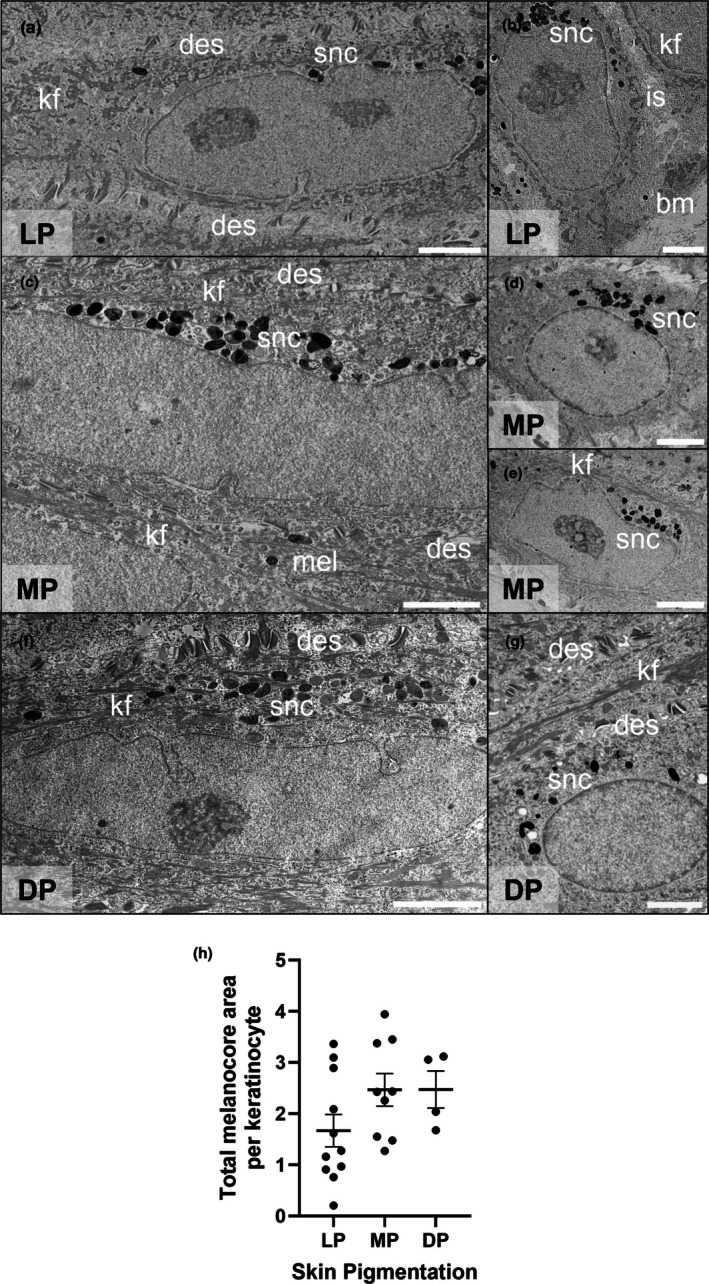
Ultrastructural analysis of melanin distribution in keratinocytes of human pigmented full‐thickness skin equivalents. Representative TEM micrographs of sectioned human pigmented skin equivalents showing the formation of the melanin supranuclear cap (snc) keratinocytes in different layers of the epidermis: (a) *Stratum spinosum* and (b) *stratum basale* keratinocytes from lightly pigmented skin equivalents (LP); (c) *Stratum granulosum* and (d, e) *stratum spinosum* keratinocytes from moderately pigmented skin equivalents (MP); (f) *Stratum granulosum* and (g) *stratum spinosum* keratinocytes from darkly pigmented skin equivalents (DP). (h) Total melanokerasome area per keratinocyte (μm^2^). GraphPad Prism software was used to generate the scatter plot graphs displaying the individual values in each group and mean ± SD. LP: 124 melanokerasomes, 11 keratinocytes; MP: 195 melanokerasomes, 9 keratinocytes; DP: 109 melanokerasomes, 4 keratinocytes. Key: basement membrane (bm), desmosomes (des), intercellular space (is), keratin filaments (kf), melanokerasomes (mel), supranuclear cap (snc). Scale bars: 2 μm.

Supranuclear caps were present in the keratinocytes at different layers in all the PSEs tested (Figure 6). The formation of a SNC is essential for the role of melanin in photoprotection and is located apical to the nucleus, acting to reduce damaging UVR from reaching keratinocyte DNA (Brenner & Hearing, 2008; Kobayashi et al., 1998). SNCs within the keratinocytes of PSEs were apical to the nuclei as expected (Figure 6a–g). Electron‐dense desmosome proteins were identified spanning the intercellular space and delimiting the keratinocytes with keratin filaments, aiding the identification of keratinocytes within the multi‐cellular tissue construct. As observed by histological staining (Figure 1d), SNCs were more visible in darker PSEs. Keratinocytes within the lower epidermal layers were identified by a more rounded nucleus with melanokerasomes crowding over the apical surface of the nucleus (Figure 6b,d,e,g). In contrast, flattened keratinocytes present in the upper epidermal layers displayed extended SNCs that were evident in moderately and darkly PSEs (Figure 6c,f), whereas lightly PSEs demonstrated only a few sparse melanokerasomes (Figure 6a). Preliminary morphometric data demonstrated that the total area of melanokerasomes that formed SNCs in keratinocytes were smaller in more lightly PSEs (Figure 6h).

**FIGURE 7:**
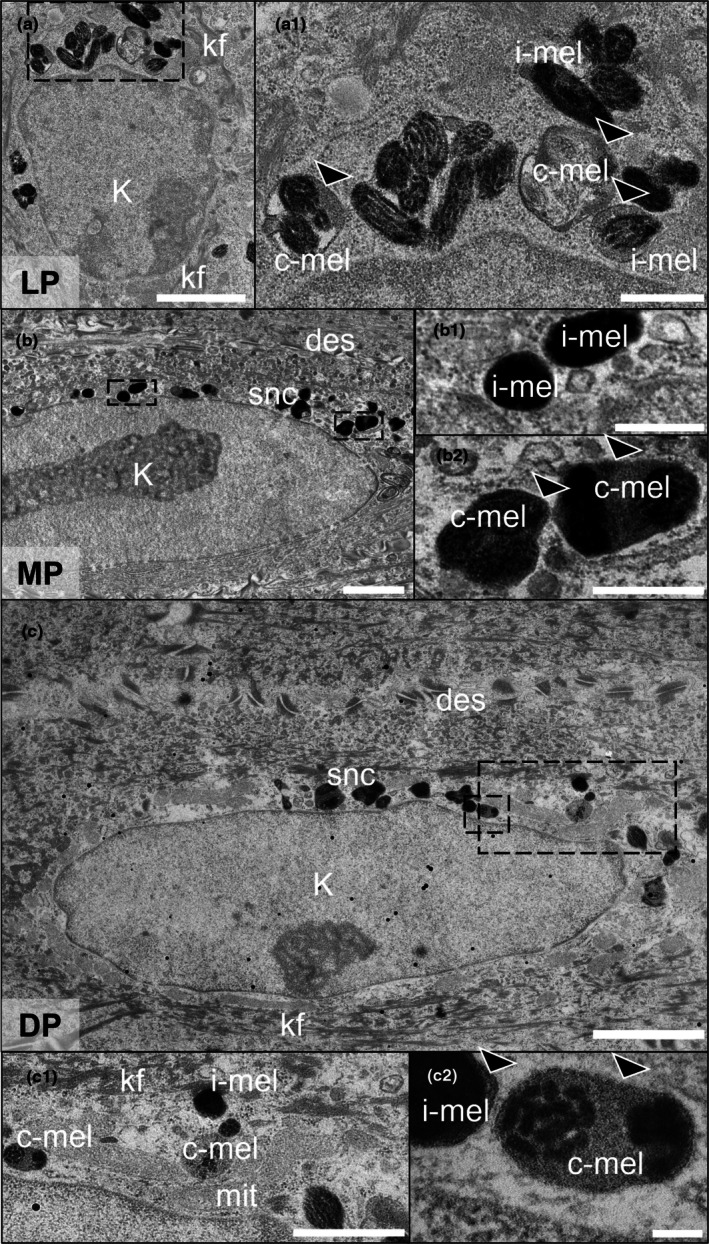
Melanin granules internalised by keratinocytes in human pigmented full‐thickness skin equivalents are present in single membrane‐bound compartments that contain isolated and clustered melanokerasomes. Representative TEM micrographs of sectioned human pigmented skin equivalents. Keratinocyte showing organisation of supranuclear melanin cap in lightly (a), moderately (b) and darkly (c) pigmented skin equivalents (LP, MP, DP, respectively). The boxed regions in A, B and C are shown at a higher magnification in a1, b1, c1 and c2. Melanin granules and mitochondria (mit) are shown above the keratinocyte nucleus. High magnification micrograph of melanokerasomes in the supranuclear cap containing an isolated melanokerasome (i‐mel) and a cluster of melanokerasomes (c‐mel) surrounded by a single membrane (arrowheads). Electro‐dense desmosome junctions (des) spanning between keratinocytes (K). Keratin filaments (kf). Scale bars: 2 μm (a, b, c), 500 nm (a1, b1, b2), 100 nm (c1, c2).

We also examined the distribution of melanokerasomes inside the SNCs generated in vitro (Figure 7). Different melanin organisation was observed in keratinocytes representative of different skin tones in vitro, which correlates with findings reported in human skin in vivo (Hurbain et al., 2018). Qualitative observations of ultra‐thin sections examined via electron microscopy suggest there could be differences in melanin granule organisation between the bioengineered PSE constructs. Clusters of melanokerasomes, which are groups of melanocores surrounded by a single outer membrane and embedded within a heterogenous granular matrix, and isolated melanokerasomes have both been identified in SNCs *in vivo* (Hurbain et al., 2018). We observed that PSEs containing keratinocytes co‐cultured with melanocytes from different skin tones possessed these two types of melanin organisation: isolated melanokerasomes (i‐mel) and clustered melanokerasomes (c‐mel) surrounded by a single membrane (Figure 7a1, b1, b2, c1, c2). Qualitatively, it appears that basal keratinocytes from lightly PSEs displayed larger clusters of melanokerasomes (Figures 6b and 7a1) compared to those found at different epidermal layers in moderately and darkly PSEs. In addition, the presence of mitochondria close to the clusters of melanokerasomes was noted during the characterisation of keratinocytes (Figure 6c1). This has been proposed to have a role in expanding the surface area of the melanin‐containing SNC structures and the mobilisation of melanokerasomes when under UV stress, thus enabling increased capping efficiency compared to isolated melanokerasomes (Hurbain et al., 2018).

## 4 DISCUSSION

In this study, we have provided evidence that anatomical differences in skin tones found in native human skin can be recreated in vitro. We have successfully generated novel PSEs exhibiting features found in light, moderate and dark skin phenotypes. Together with detailed morphological analysis, this represents a significant advance in the ability to create skin models relevant to the study of human skin pigmentation. The generation of different skin phenotypes has been reported in some epidermal equivalents (Bernerd et al., 2012; Bessou et al., 1996) and full‐thickness skin equivalents (Böttcher‐Haberzeth et al., 2013; Duval et al., 2012; Hedley et al., 2002). However, such approaches have suffered from various limitations such as the absence or lack of a physiologically representative dermal compartment, which ultimately can impact the structure and function of melanin within the tissue. The correct localisation and, therefore, protective nature of melanin within the epidermis has been linked to dermo‐epidermal interactions through several mechanisms: biochemical ECM interactions, direct cell–cell contact, secretion of paracrine mediators and basement membrane formation (Cario‐André et al., 2006; Duval et al., 2014; Hedley et al., 2002; Lee et al., 2003; Minwalla et al., 2001; Yoshida et al., 2007). Moreover, the lack of a dermal compartment, the inclusion of exogenous ECM proteins into the dermal compartment, unknown murine factors present in skin xenografts or the use of de‐epidermised acellular materials can introduce additional variables that impact the ability to bioengineer a physiologically representative human dermal microenvironment required to recapitulate the complex structure and function of human skin (Cario‐André et al., 2006; Duval et al., 2014; El Ghalbzouri et al., 2005, 2009). The approach described within this study relies on a dermal foundation populated by human fibroblasts that neo‐synthesise a complex network of ECM proteins and signalling molecules that better resembles native skin tissue. This, in conjunction with the inclusion of primary melanocytes derived from alternative skin tones, enabled us to create corresponding PSEs in vitro. Herein, we demonstrate how these novel complex human skin models can be used to provide valuable fundamental insights into melanin dynamics within the tissue that would otherwise be difficult to achieve through invasive biopsy collection.

Macroscopic and histological analyses showed that bioengineered human PSEs exhibited significant differences in appearance and melanin distribution across alternative skin tones. ITA classification confirmed that the generated PSEs objectively resembled the values expected for native human pigmentation (Del Bino et al., 2006; Del Bino & Bernerd, 2013). Our findings are particularly potent due to the combination of Caucasian cells (keratinocytes and fibroblasts) with melanocytes of varying phototypes. This emphasises the integral role of melanocytes in the determination of constitutive pigmentation and provides a unique platform to further probe the contribution of keratinocyte and fibroblast origin in melanin homeostasis. A distinct feature of the PSEs described herein is the formation of the SNCs within keratinocytes. Differences in melanin expression between skin tones were directly comparable to those found in native skin in vivo (Del Bino et al., 2015; Del Bino & Bernerd, 2013). Histological staining and immunofluorescence techniques are well‐established approaches for assessing epidermal pigmentation and melanocyte differentiation markers in epidermal and full‐thickness skin equivalents (Bessou et al., 1996; Cario‐André et al., 2000; Duval et al., 2012; Gibbs et al., 2000; Goyer et al., 2019; Hedley et al., 2002; Liu et al., 2007; Yoshida et al., 2007). In addition to these approaches, we have also performed in‐depth ultrastructural analyses using TEM, a technique absent in the characterisation of many bioengineered PSEs (Choi et al., 2010; Gledhill et al., 2015; Yamaguchi et al., 2006). This approach has revealed the ultrastructural changes associated with melanosome biogenesis, maturation, transfer and melanin organisation, which are comparable to the processes reported in vivo (Daniele et al., 2014; Delevoye et al., 2009; Domingues et al., 2020; Ebanks et al., 2011; Hurbain et al., 2008; Loubéry et al., 2012; Minwalla et al., 2001; Setty et al., 2008; Thong et al., 2003).

We have demonstrated the use of our functional PSE system to provide insight into the dynamics that govern melanin distribution and how this differs across skin tones. Ultrastructural features of melanosome biogenesis and maturation were demonstrated through TEM analysis of our in vitro generated skin tone palette. A variety of in vitro studies have described melanosome development in 2D cell systems (Delevoye et al., 2009; Hurbain et al., 2008; Loubéry et al., 2012; Setty et al., 2008), but only a handful of studies have reported melanocytes localised in the *stratum basale* (Bessou et al., 1996; Gibbs et al., 2000; Goyer et al., 2019; Hall et al., 2022; Liu et al., 2007), and identification of melanosome stages in PSEs (Goyer et al., 2019; Yoshida et al., 2007). Unique to this study, we have further characterised melanosome staged development across different human skin tones in vitro and described the size and shape of melanosomes in melanocytes, which have not been previously delineated. We report that melanosome size and shape patterns correspond to the overall level of pigmentation, with large differences observed between lighter and darker skin conditions. Although previous studies have described melanosome size in vitro, these have been limited to 2D culture systems (Minwalla et al., 2001; Thong et al., 2003), which are inherently deficient systems at predicting morphometric parameters due to the flattened shape adopted by cells poorly representing human skin. Similarly, organelle shape descriptors such as Feret diameter and circularity have been described for mitochondria analysis (Faitg et al., 2020), but not previously applied to the context of melanosomes. Herein, we combined a detailed, ultrastructural, morphological analysis with physiologically representative human PSEs that generated robust data relevant to melanosome dynamics in vivo.

Within developed PSEs, there was a noticeable correlation between the presence of mitochondria and melanosomes, which is supported by previous studies suggestive that melanosome‐mitochondrion physical interactions are relevant for organelle biogenesis and changes in pigmentation (Daniele et al., 2014). The PSEs reported herein could represent a platform to study mitochondria and melanosome crosstalk and provide new insights into melanin regulation and mitochondrial function (Kaushik et al., 2023; Kim et al., 2014). Furthermore, although some studies in human skin describe melanosome anatomy and physiology (Daniele et al., 2014; Delevoye et al., 2009; Domingues et al., 2020; Hurbain et al., 2008; Le et al., 2021; Raposo & Marks, 2007; Thong et al., 2003), there is less information about the morphology and quantification data from melanosomes in melanocytes. Our findings emphasise the diverse research applications that this PSE platform provides.

We also describe the presence of mature melanosomes in dendrites between epidermal keratinocytes. In vivo, mature melanosomes are translocated along microtubules to actin‐rich dendritic tips (Loubéry et al., 2012) and then transferred to keratinocytes. To our knowledge, morphometric analysis of melanosomes in dendrites in vitro and in vivo has not yet been reported.

One of the most recognised differences between pigmentation phenotypes in native human skin is the difference in the quantity of melanin synthesised and transferred to keratinocytes. An established mechanism of melanin transfer is the exocytosis/phagocytosis model, in which melanin granules are released from melanocyte dendrites to the extracellular space and internalised by keratinocytes (Hurbain et al., 2018; Moreiras et al., 2022; Tarafder et al., 2014). In support of this model, our data provide evidence of melanocores in the extracellular space between melanocyte dendrites and keratinocytes and examples of melanocores fusing with the keratinocyte plasma membrane. Differences in melanin content, SNC size and melanin distribution in keratinocytes across the epidermal layers have been identified between pigmentation phenotypes in vivo (Alaluf et al., 2003; Del Bino et al., 2015; Glimcher et al., 1973; Hurbain et al., 2018; Szabo et al., 1969; Tadokoro et al., 2005). Our bioengineered human PSEs showed a similar formation of SNCs and their variation between epidermal layers across alternative skin tones. Preliminary TEM data depicting melanokerasome area per keratinocyte per skin tone correlated well with the size of SNCs visibly identified by standard histological methods, as the total melanokerasome area in keratinocytes within the lightly PSEs is smaller than in darker PSEs. In addition, melanokerasome organisation and distribution in the upper and lower epidermis of PSEs correlated with studies of skin tones in native skin (Hurbain et al., 2018), which is thought to be regulated by keratinocytes (Minwalla et al., 2001; Yoshida et al., 2007). The level of ultrastructural analyses performed in this study not only confirm the presence of melanokerasomes but also provide detailed morphological information about melanokerasome features in keratinocytes in human skin *in vitro*. To our knowledge, such detail has not been described before, highlighting the potential applications of this technology for skin pigmentation research.

In this study, we have developed a series of bespoke PSEs to investigate the intricacies that govern skin pigmentation and melanin dynamics in vitro. We recreated skin tone phenotypes in vitro and characterised them to demonstrate melanin structures similar to those found in human native skin. Due to their spatiotemporal nature, melanosome formation, transfer and organisation are often challenging to study in vitro and in vivo. However, the novel PSEs described herein offer a valuable and accessible alternative to follow such dynamic processes, which are physiologically representative within the context of pigmentation, sharing many characteristics similar to those of pigmented skin in vivo. Moreover, such models enable investigations of this nature over time, reducing the reliance on extensive clinical tests and will be valuable for applications ranging from cosmetic testing to diverse aspects of skin pigmentation research.

## 5 CONCLUSIONS

In this study, we describe the increased complexity of our human skin platform through the incorporation of melanocytes representative of diverse skin tones. We demonstrate that ultrastructure at a subcellular level compares favourably to in vivo pigmented skin diversity and allows the study of the differential microanatomy of skin pigmentation processes. We present proof‐of‐concept data that ultrastructural morphometric changes and defined patterns among skin tones correlate to the pigmentation level of the PSEs. This represents a valuable tool to study fundamental mechanisms that remain elusive and govern constitutive pigmentation. Potential applications of this platform include fundamental research into skin pigmentation, research and development applications such as the design and screening of actives that modulate pigmentation either as single molecular entities or whole formulations, and opportunities to model and investigate skin pigmentation disorders related to complex skin conditions.

## AUTHOR CONTRIBUTIONS

PD: Conceptualisation, data curation, formal analysis, investigation, methodology, visualisation and writing—original draft. RR: Formal Analysis, investigation and writing—review and editing. PR: Methodology and writing—review and editing. LS: Methodology and writing—review and editing. AS: Methodology and writing—review and editing. KG: Visualisation and writing—review and editing. SP: Conceptualisation, funding acquisition, project administration, supervision and writing—review and editing.

## FUNDING INFORMATION

This work was supported by a training studentship from the Mexican National Council of Science and Technology (CONACyT) grant number: 2019‐000021‐01‐EXTF‐00262, the Biotechnology and Biological Sciences Research Council (BBSRC) grant number BB/S007431/1 and The Anatomical Society.

## CONFLICT OF INTEREST STATEMENT

Author SP collaborates and acts as a technical consultant for company Reprocell Europe Ltd. The remaining authors have no conflicts of interest to declare.

## ETHICS STATEMENT

No animal materials were used in this study. Human bioengineered skin equivalents were produced using only commercially available established cell lines.

## Data Availability

The data that support the findings presented in the figures shown are available from the corresponding author upon reasonable request.

## References

[joa70026-bib-0001] Abaci, H.E. , Coffman, A. , Doucet, Y. , Chen, J. , Jacków, J. , Wang, E. et al. (2018) Tissue engineering of human hair follicles using a biomimetic developmental approach. Nature Communications, 9(1), 1–11. Available from: 10.1038/s41467-018-07579-y PMC6294003 30546011

[joa70026-bib-0002] Alaluf, S. , Barrett, K. , Blount, M. & Carter, N. (2003) Ethnic variation in tyrosinase and TYRP1 expression in photoexposed and photoprotected human skin. Pigment Cell Research, 16(1), 35–42. Available from: 10.1034/j.1600-0749.2003.00005.x 12519123

[joa70026-bib-0003] Ando, H. , Niki, Y. , Ito, M. , Akiyama, K. , Matsui, M.S. , Yarosh, D.B. et al. (2012) Melanosomes are transferred from melanocytes to keratinocytes through the processes of packaging, release, uptake, and dispersion. Journal of Investigative Dermatology, 132(4), 1222–1229. Available from: 10.1038/jid.2011.413 22189785

[joa70026-bib-0004] Bechetoille, N. , Dezutter‐Dambuyant, C. , Damour, O. , André, V. , Orly, I. & Perrier, E. (2007) Effects of solar ultraviolet radiation on engineered human skin equivalent containing both langerhans cells and dermal dendritic cells. Tissue Engineering, 13(11), 2667–2679. Available from: 10.1089/ten.2006.0405 17883323

[joa70026-bib-0005] Bernerd, F. , Marionnet, C. & Duval, C. (2012) Solar ultraviolet radiation induces biological alterations in human skin in vitro: relevance of a well‐balanced UVA/UVB protection. Indian Journal of Dermatology, Venereology and Leprology, 78(SUPPL.1), 15–23. Available from: 10.4103/0378-6323.97351 22710109

[joa70026-bib-0006] Bessou, S. , Surlève‐Bazeille, J.E. , Pain, C. , Donatien, P. & Taïeb, A. (1996) Ex vivo study of skin phototypes. Journal of Investigative Dermatology, 107(5), 684–688. Available from: 10.1111/1523-1747.ep12365574 8875949

[joa70026-bib-0007] Böttcher‐Haberzeth, S. , Klar, A.S. , Biedermann, T. , Schiestl, C. , Meuli‐Simmen, C. , Reichmann, E. et al. (2013) “Trooping the color”: restoring the original donor skin color by addition of melanocytes to bioengineered skin analogs. Pediatric Surgery International, 29(3), 239–247. Available from: 10.1007/s00383-012-3217-0 23196807

[joa70026-bib-0008] Bourland, J. , Fradette, J. & Auger, F.A. (2018) Tissue‐engineered 3D melanoma model with blood and lymphatic capillaries for drug development. Scientific Reports, 8(1), 1–13. Available from: 10.1038/s41598-018-31502-6 30181613 PMC6123405

[joa70026-bib-0009] Brauchle, E. , Johannsen, H. , Nolan, S. , Thude, S. & Schenke‐Layland, K. (2013) Design and analysis of a squamous cell carcinoma in vitro model system. Biomaterials, 34(30), 7401–7407. Available from: 10.1016/j.biomaterials.2013.06.016 23827189

[joa70026-bib-0010] Brenner, M. & Hearing, V.J. (2008) The protective role of melanin against UV damage in human skin. Photochemistry and Photobiology, 84(3), 539–549. Available from: 10.1111/j.1751-1097.2007.00226.x 18435612 PMC2671032

[joa70026-bib-0011] Cario‐André, M. , Pain, C. , Gauthier, Y. , Casoli, V. & Taieb, A. (2006) In vivo and in vitro evidence of dermal fibroblasts influence on human epidermal pigmentation. Pigment Cell Research, 19(5), 434–442. Available from: 10.1111/j.1600-0749.2006.00326.x 16965272

[joa70026-bib-0012] Cario‐André, M. , Pain, C. , Taïeb, A. , Nikaido, O. , Gall, Y. & Ginestar, J. (2000) Studies on epidermis reconstructed with and without melanocytes: melanocytes prevent sunburn cell formation but not appearance of DNA damaged cells in fair‐skinned caucasians. Journal of Investigative Dermatology, 115(2), 193–199. Available from: 10.1046/j.1523-1747.2000.00007.x 10951235

[joa70026-bib-0013] Cario‐André, M. , Pain, C. , Gauthier, Y. & Taïeb, A. (2007) The melanocytorrhagic hypothesis of vitiligo tested on pigmented, stressed, reconstructed epidermis. Pigment Cell Research, 20(5), 385–393. Available from: 10.1111/j.1600-0749.2007.00396.x 17850512

[joa70026-bib-0014] Choi, W. , Wolber, R. , Gerwat, W. , Mann, T. , Batzer, J. , Smuda, C. et al. (2010) The fibroblast‐derived paracrine factor neuregulin‐1 has a novel role in regulating the constitutive color and melanocyte function in human skin. Journal of Cell Science, 123(18), 3102–3111. Available from: 10.1242/jcs.064774 20736300 PMC2931604

[joa70026-bib-0015] Daniele, T. , Hurbain, I. , Vago, R. , Casari, G. , Raposo, G. , Tacchetti, C. et al. (2014) Mitochondria and melanosomes establish physical contacts modulated by Mfn2 and involved in organelle biogenesis. Current Biology, 24(4), 393–403. Available from: 10.1016/j.cub.2014.01.007 24485836

[joa70026-bib-0016] De Los Santos Gomez, P. , Costello, L. , Goncalves, K. & Przyborski, S. (2024) Comparison of photodamage in non‐pigmented and pigmented human skin equivalents exposed to repeated ultraviolet radiation to investigate the role of melanocytes in skin photoprotection. Frontiers in Medicine, 11, 1355799. Available from: 10.3389/fmed.2024.1355799 38698778 PMC11063240

[joa70026-bib-0017] Del Bino, S. & Bernerd, F. (2013) Variations in skin colour and the biological consequences of ultraviolet radiation exposure. British Journal of Dermatology, 169(Suppl. 3), 33–40. Available from: 10.1111/bjd.12529 24098899

[joa70026-bib-0018] Del Bino, S. , Ito, S. , Sok, J. , Nakanishi, Y. , Bastien, P. , Wakamatsu, K. et al. (2015) Chemical analysis of constitutive pigmentation of human epidermis reveals constant eumelanin to pheomelanin ratio. Pigment Cell and Melanoma Research, 28(6), 707–717. Available from: 10.1111/pcmr.12410 26285058

[joa70026-bib-0019] Del Bino, S. , Sok, J. , Bessac, E. & Bernerd, F. (2006) Relationship between skin response to ultraviolet exposure and skin color type. Pigment Cell Research, 19(6), 606–614. Available from: 10.1111/j.1600-0749.2006.00338.x 17083487

[joa70026-bib-0020] Delevoye, C. , Hurbain, I. , Tenza, D. , Sibarita, J.B. , Uzan‐Gafsou, S. , Ohno, H. et al. (2009) AP‐1 and KIF13A coordinate endosomal sorting and positioning during melanosome biogenesis. Journal of Cell Biology, 187(2), 247–264. Available from: 10.1083/jcb.200907122 19841138 PMC2768840

[joa70026-bib-0021] Domingues, L. , Hurbain, I. , Gilles‐Marsens, F. , Sirés‐Campos, J. , André, N. , Dewulf, M. et al. (2020) Coupling of melanocyte signaling and mechanics by caveolae is required for human skin pigmentation. Nature Communications, 11(1), 2988. Available from: 10.1038/s41467-020-16738-z PMC7293304 32532976

[joa70026-bib-0022] Duval, C. , Chagnoleau, C. , Pouradier, F. , Sextius, P. , Condom, E. & Bernerd, F. (2012) Human skin model containing melanocytes: essential role of keratinocyte growth factor for constitutive pigmentation‐functional response to α‐melanocyte stimulating hormone and forskolin. Tissue Engineering ‐ Part C: Methods, 18(12), 947–957. Available from: 10.1089/ten.tec.2011.0676 22646688

[joa70026-bib-0023] Duval, C. , Cohen, C. , Chagnoleau, C. , Flouret, V. , Bourreau, E. & Bernerd, F. (2014) Key regulatory role of dermal fibroblasts in pigmentation as demonstrated using a reconstructed skin model: impact of photo‐aging. PLoS One, 9(12), 1–25. Available from: 10.1371/journal.pone.0114182 PMC4260844 25490395

[joa70026-bib-0024] Duval, C. , Régnier, M. & Schmidt, R. (2001) Distinct melanogenic response of human melanocytes in mono‐culture, in co‐culture with keratinocytes and in reconstructed epidermis, to UV exposure. Pigment Cell Research, 14(5), 348–355. Available from: 10.1034/j.1600-0749.2001.140506.x 11601656

[joa70026-bib-0025] Duval, C. , Schmidt, R. , Regnier, M. , Facy, V. , Asselineau, D. & Bernerd, F. (2003) The use of reconstructed human skin to evaluate UV‐induced modifications and sunscreen efficacy. Experimental Dermatology, 12, 64–70. Available from: 10.1034/j.1600-0625.12.s2.10.x 14756526

[joa70026-bib-0026] Ebanks, J.P. , Koshoffer, A. , Wickett, R.R. , Schwemberger, S. , Babcock, G. , Hakozaki, T. et al. (2011) Epidermal keratinocytes from light vs. dark skin exhibit differential degradation of melanosomes. Journal of Investigative Dermatology, 131(6), 1226–1233. Available from: 10.1038/jid.2011.22 21326292

[joa70026-bib-0027] El Ghalbzouri, A. , Commandeur, S. , Rietveld, M.H. , Mulder, A.A. & Willemze, R. (2009) Replacement of animal‐derived collagen matrix by human fibroblast‐derived dermal matrix for human skin equivalent products. Biomaterials, 30(1), 71–78. Available from: 10.1016/j.biomaterials.2008.09.002 18838164

[joa70026-bib-0028] El Ghalbzouri, A. , Jonkman, M.F. , Dijkman, R. & Ponec, M. (2005) Basement membrane reconstruction in human skin equivalents is regulated by fibroblasts and/or exogenously activated keratinocytes. Journal of Investigative Dermatology, 124(1), 79–86. Available from: 10.1111/j.0022-202X.2004.23549.x 15654956

[joa70026-bib-0029] Faitg, J. , Davey, T. , Turnbull, D.M. , White, K. & Vincent, A.E. (2020) Mitochondrial morphology and function: two for the price of one! Journal of Microscopy, 278(2), 89–106. Available from: 10.1111/jmi.12891 32277765

[joa70026-bib-0030] Freer, M. , Darling, N. , Goncalves, K. , Mills, K.J. & Przyborski, S. (2023) Development of a mammalian neurosensory full‐thickness skin equivalent and its application to screen sensitizing stimuli. Bioengineering and Translational Medicine, 8(3), 1–13. Available from: 10.1002/btm2.10484 PMC10189474 37206205

[joa70026-bib-0031] Gibbs, S. , Murli, S. , De Boer, G. , Mulder, A.A. , Mommaas, A.M. & Ponec, M. (2000) Melanosome capping of keratinocytes in pigmented reconstructed epidermis—effect of ultraviolet radiation and 3‐isobutyl‐1‐methyl‐xanthine on melanogenesis. Pigment Cell Research, 13(6), 458–466. Available from: 10.1034/j.1600-0749.2000.130608.x 11153698

[joa70026-bib-0032] Gledhill, K. , Guo, Z. , Umegaki‐Arao, N. , Higgins, C.A. , Itoh, M. & Christiano, A.M. (2015) Melanin transfer in human 3D skin equivalents generated exclusively from induced pluripotent stem cells. PLoS One, 10(8), 1–16. Available from: 10.1371/journal.pone.0136713 PMC4550351 26308443

[joa70026-bib-0033] Glimcher, M.E. , Kostick, R.M. & Szabo, G. (1973) The epidermal melanocyte system in newborn human skin. A quantitative histologic study. Journal of Investigative Dermatology, 61(6), 344–347. Available from: 10.1111/1523-1747.ep12676618 4796533

[joa70026-bib-0034] Goncalves, K. , De Los Santos Gomez, P. , Costello, L. , Smith, L. , Mead, H. , Simpson, A. et al. (2023) Investigation into the effect of skin tone modulators and exogenous stress on skin pigmentation utilizing a novel bioengineered skin equivalent. Bioengineering & Translational Medicine, 8(2), 1–14. Available from: 10.1002/btm2.10415 PMC10013773 36925688

[joa70026-bib-0035] Goyer, B. , Pereira, U. , Magne, B. , Larouche, D. , Kearns‐Turcotte, S. , Rochette, P.J. et al. (2019) Impact of ultraviolet radiation on dermal and epidermal DNA damage in a human pigmented bilayered skin substitute. Journal of Tissue Engineering and Regenerative Medicine, 13(12), 2300–2311. Available from: 10.1002/term.2959 31502756

[joa70026-bib-0036] Guo, Z. , Tong, C.K. , Jacków, J. , Doucet, Y.S. , Abaci, H.E. , Zeng, W. et al. (2022) Engineering human skin model innervated with itch sensory neuron‐like cells differentiated from induced pluripotent stem cells. Bioengineering & Translational Medicine, 7(1), 2–9. Available from: 10.1002/btm2.10247 PMC8780951 35111948

[joa70026-bib-0037] Hall, M.J. , Lopes‐Ventura, S. , Neto, M.V. , Charneca, J. , Zoio, P. , Seabra, M.C. et al. (2022) Reconstructed human pigmented skin/epidermis models achieve epidermal pigmentation through melanocore transfer. Pigment Cell and Melanoma Research, 35(4), 425–435. Available from: 10.1111/pcmr.13039 35325505 PMC9543140

[joa70026-bib-0038] Hardwick, R.N. , Betts, C.J. , Whritenour, J. , Sura, R. , Thamsen, M. , Kaufman, E.H. et al. (2020) Drug‐induced skin toxicity: gaps in preclinical testing cascade as opportunities for complex: in vitro models and assays. Lab on a Chip, 20(2), 199–214. Available from: 10.1039/c9lc00519f 31598618

[joa70026-bib-0039] Hedley, S.J. , Layton, C. , Heaton, M. , Chakrabarty, K.H. , Dawson, R.A. , Gawkrodger, D.J. et al. (2002) Fibroblasts play a regulatory role in the control of pigmentation in reconstructed human skin from skin types I and II. Pigment Cell Research, 15(1), 49–56. Available from: 10.1034/j.1600-0749.2002.00067.x 11837456

[joa70026-bib-0040] Hofmann, E. , Fink, J. , Pignet, A.‐L. , Schwarz, A. , Schellnegger, M. , Nischwitz, S.P. et al. (2023) Human in vitro skin models for wound healing and wound healing disorders. Biomedicine, 11(4), 1056. Available from: 10.3390/biomedicines11041056 PMC10135654 37189674

[joa70026-bib-0041] Hofmann, E. , Schwarz, A. , Fink, J. , Kamolz, L.‐P. & Kotzbeck, P. (2023) Modelling the complexity of human skin in vitro. Biomedicine MDPI, 11, 794. Available from: 10.3390/biomedicines11030794 PMC10045055 36979772

[joa70026-bib-0042] Hurbain, I. , Geerts, W.J.C. , Boudier, T. , Marco, S. , Verkleij, A.J. , Marks, M.S. et al. (2008) Electron tomography of early melanosomes: implications for melanogenesis and the generation of fibrillar amyloid sheets. Proceedings of the National Academy of Sciences, 105, 19726–19731. Available from: www.pnas.org/cgi/content/full/ 10.1073/pnas.0803488105 PMC2604932 19033461

[joa70026-bib-0043] Hurbain, I. , Romao, M. , Sextius, P. , Bourreau, E. , Marchal, C. , Bernerd, F. et al. (2018) Melanosome distribution in keratinocytes in different skin types: melanosome clusters are not degradative organelles. Journal of Investigative Dermatology, 138(3), 647–656. Available from: 10.1016/j.jid.2017.09.039 29054596

[joa70026-bib-0044] Jang, H.J. , Lee, J.B. & Yoon, J.K. (2023) Advanced in vitro three‐dimensional skin models of atopic dermatitis. Tissue Engineering and Regenerative Medicine, 20(4), 539–552. Available from: 10.1007/s13770-023-00532-1 36995643 PMC10313606

[joa70026-bib-0045] Kaushik, H. , Kumar, V. & Parsad, D. (2023) Mitochondria–melanocyte cellular interactions: an emerging mechanism of vitiligo pathogenesis. Journal of the European Academy of Dermatology and Venereology, 37, 2196–2207. Available from: 10.1111/jdv.19019 36897230

[joa70026-bib-0046] Kim, B.S. , Gao, G. , Kim, J.Y. & Cho, D.W. (2019) 3D cell printing of Perfusable vascularized human skin equivalent composed of epidermis, dermis, and hypodermis for better structural recapitulation of native skin. Advanced Healthcare Materials, 8(7), 1–11. Available from: 10.1002/adhm.201801019 30358939

[joa70026-bib-0047] Kim, E.S. , Park, S.J. , Goh, M.J. , Na, Y.J. , Jo, D.S. , Jo, Y.K. et al. (2014) Mitochondrial dynamics regulate melanogenesis through proteasomal degradation of MITF via ROS‐ERK activation. Pigment Cell and Melanoma Research, 27(6), 1051–1062. Available from: 10.1111/pcmr.12298 25065405

[joa70026-bib-0048] Kobayashi, N. , Nakagawa, A. , Muramatsu, T. , Yamashina, Y. , Shirai, T. , Hashimoto, M.W. et al. (1998) Supranuclear melanin caps reduce ultraviolet induced DNA photoproducts in human epidermis. Journal of Investigative Dermatology, 110(5), 806–810. Available from: 10.1046/j.1523-1747.1998.00178.x 9579550

[joa70026-bib-0049] Kosten, I.J. , Spiekstra, S.W. , de Gruijl, T.D. & Gibbs, S. (2015) MUTZ‐3 derived Langerhans cells in human skin equivalents show differential migration and phenotypic plasticity after allergen or irritant exposure. Toxicology and Applied Pharmacology, 287(1), 35–42. Available from: 10.1016/j.taap.2015.05.017 26028481

[joa70026-bib-0050] Le, L. , Sirés‐Campos, J. , Raposo, G. , Delevoye, C. & Marks, M.S. (2021) Melanosome biogenesis in the pigmentation of mammalian skin. Integrative and Comparative Biology, 61(4), 1517–1545. Available from: 10.1093/icb/icab078 34021746 PMC8516112

[joa70026-bib-0051] Lee, D. , Lee, D.Y. , Lee, J.H. , Lee, E.S. , Cho, K.H. & Yang, J.M. (2003) Fibroblasts play a stimulatory role in keratinocyte proliferation but an inhibitory role in melanocyte growth and pigmentation in a skin equivalent system from skin type IV. Archives of Dermatological Research, 294(10), 444–446. Available from: 10.1007/s00403-002-0359-2 12563542

[joa70026-bib-0052] Lejeune, F. , Christiaens, F. & Bernerd, F. (2008) Evaluation of sunscreen products using a reconstructed skin model exposed to simulated daily ultraviolet radiation: relevance of filtration profile and SPF value for daily photoprotection. Photodermatology, Photoimmunology & Photomedicine, 24(5), 249–255. Available from: 10.1111/j.1600-0781.2008.00370.x 18811866

[joa70026-bib-0053] Liu, Y. , Suwa, F. , Wang, X. , Takemura, A. , Fang, Y.R. , Li, Y. et al. (2007) Reconstruction of a tissue‐engineered skin containing melanocytes. Cell Biology International, 31(9), 985–990. Available from: 10.1016/j.cellbi.2007.03.009 17467308

[joa70026-bib-0054] Loubéry, S. , Delevoye, C. , Louvard, D. , Raposo, G. & Coudrier, E. (2012) Myosin VI regulates actin dynamics and melanosome biogenesis. Traffic, 13(5), 665–680. Available from: 10.1111/j.1600-0854.2012.01342.x 22321127

[joa70026-bib-0055] Michalczyk, T. , Biedermann, T. , Böttcher‐Haberzeth, S. , Klar, A.S. , Meuli, M. & Reichmann, E. (2018) UVB exposure of a humanized skin model reveals unexpected dynamic of keratinocyte proliferation and Wnt inhibitor balancing. Journal of Tissue Engineering and Regenerative Medicine, 12(2), 505–515. Available from: 10.1002/term.2519 28715139

[joa70026-bib-0056] Minwalla, L. , Zhao, Y. , Boissy, R.E. , le Poole, I.C. & Wickett, R.R. (2001) Keratinocytes play a role in regulating distribution patterns of recipient melanosomes in vitro. Journal of Investigative Dermatology, 117(2), 341–347. Available from: 10.1046/j.0022-202X.2001.01411.x 11511313

[joa70026-bib-0057] Moreiras, H. , Bento‐Lopes, L. , Neto, M.V. , Escrevente, C. , Cabaço, L.C. , Hall, M.J. et al. (2022) Melanocore uptake by keratinocytes occurs through phagocytosis and involves protease‐activated receptor‐2 internalization. Traffic, 23(6), 331–345. Available from: 10.1111/tra.12843 35426185 PMC9543991

[joa70026-bib-0058] Moreiras, H. , Pereira, F.J.C. , Neto, M.V. , Bento‐Lopes, L. , Festas, T.C. , Seabra, M.C. et al. (2020) The exocyst is required for melanin exocytosis from melanocytes and transfer to keratinocytes. Pigment Cell and Melanoma Research, 33(2), 366–371. Available from: 10.1111/pcmr.12840 31665827

[joa70026-bib-0059] Mori, N. , Morimoto, Y. & Takeuchi, S. (2017) Skin integrated with perfusable vascular channels on a chip. Biomaterials, 116, 48–56. Available from: 10.1016/j.biomaterials.2016.11.031 27914266

[joa70026-bib-0060] Neto, M.V. , Hall, M.J. , Charneca, J. , Escrevente, C. , Seabra, M.C. & Barral, D.C. (2024) Photoprotective melanin is maintained within keratinocytes in storage lysosomes. Journal of Investigative Dermatology [Preprint]. Available from: 10.1016/j.jid.2024.08.023 39303907

[joa70026-bib-0061] Rademacher, F. , Simanski, M. , Gläser, R. & Harder, J. (2018) Skin microbiota and human 3D skin models. Experimental Dermatology, 27(5), 489–494. Available from: 10.1111/exd.13517 29464787

[joa70026-bib-0062] Raposo, G. & Marks, M.S. (2007) Melanosomes—dark organelles enlighten endosomal mem. Nature Molecular Cell Biology, 8(10), 786–797. Available from: 10.1038/nrm2258.Melanosomes 17878918 PMC2786984

[joa70026-bib-0063] Rioux, G. , Simard, M. , Morin, S. , Lorthois, I. , Guérin, S.L. & Pouliot, R. (2021) Development of a 3D psoriatic skin model optimized for infiltration of IL‐17A producing T cells: focus on the crosstalk between T cells and psoriatic keratinocytes. Acta Biomaterialia, 136, 210–222. Available from: 10.1016/j.actbio.2021.09.018 34547515

[joa70026-bib-0064] Roger, M. , Fullard, N. , Costello, L. , Bradbury, S. , Markiewicz, E. , O'Reilly, S. et al. (2019) Bioengineering the microanatomy of human skin. Journal of Anatomy, 234(4), 438–455. Available from: 10.1111/joa.12942 30740672 PMC6422806

[joa70026-bib-0065] Salducci, M. , André, N. , Guéré, C. , Martin, M. , Fitoussi, R. , Vié, K. et al. (2014) Factors secreted by irradiated aged fibroblasts induce solar lentigo in pigmented reconstructed epidermis. Pigment Cell and Melanoma Research, 27(3), 502–504. Available from: 10.1111/pcmr.12234 24533682

[joa70026-bib-0066] Schindelin, J. , Arganda‐Carreras, I. , Frise, E. , Kaynig, V. , Longair, M. , Pietzsch, T. et al. (2012) Fiji: an open‐source platform for biological‐image analysis. Nature Methods, 9(7), 676–682. Available from: 10.1038/nmeth.2019 22743772 PMC3855844

[joa70026-bib-0067] Setty, S.R.G. , Tenza, D. , Sviderskaya, E.V. , Bennett, D.C. , Raposo, G. & Marks, M.S. (2008) Cell‐specific ATP7A transport sustains copper‐dependent tyrosinase activity in melanosomes. Nature, 454(7208), 1142–1146. Available from: 10.1038/nature07163 18650808 PMC2812007

[joa70026-bib-0068] Singh, S.K. , Kurfurst, R. , Nizard, C. , Schnebert, S. , Perrier, E. & Tobin, D.J. (2010) Melanin transfer in human skin cells is mediated by filopodia—a model for homotypic and heterotypic lysosome‐related organelle transfer. FASEB Journal, 24(10), 3756–3769. Available from: 10.1096/fj.10-159046 20501793

[joa70026-bib-0069] Sriram, G. , Bigliardi, P.L. & Bigliardi‐Qi, M. (2018) Full‐thickness human skin equivalent models of atopic dermatitis. In: Drug Discovery Today, pp. 367–383. Available from: 10.1007/7651_2018_163 29790095

[joa70026-bib-0070] Szabo, G. , Gerald, A.B. , Pathak, M.A. & Fitzpatrick, T.B. (1969) Racial differences in the fate of melanosomes in human epidermis. Nature, 222(5198), 1081–1082. Available from: 10.1038/2221081a0 5787098

[joa70026-bib-0071] Tadokoro, T. , Kobayashi, N. , Zmudzka, B.Z. , Ito, S. , Wakamatsu, K. , Yamaguchi, Y. et al. (2003) UV‐induced DNA damage and melanin content in human skin differing in racial/ethnic origin. The FASEB Journal, 17(9), 1177–1179. Available from: 10.1096/fj.02-0865fje 12692083

[joa70026-bib-0072] Tadokoro, T. , Yamaguchi, Y. , Batzer, J. , Coelho, S.G. , Zmudzka, B.Z. , Miller, S.A. et al. (2005) Mechanisms of skin tanning in different racial/ethnic groups in response to ultraviolet radiation. Journal of Investigative Dermatology, 124(6), 1326–1332. Available from: 10.1111/j.0022-202X.2005.23760.x 15955111

[joa70026-bib-0073] Tarafder, A.K. , Bolasco, G. , Correia, M.S. , Pereira, F.J.C. , Iannone, L. , Hume, A.N. et al. (2014) Rab11b mediates melanin transfer between donor melanocytes and acceptor keratinocytes via coupled exo/endocytosis. Journal of Investigative Dermatology, 134(4), 1056–1066. Available from: 10.1038/jid.2013.432 24141907

[joa70026-bib-0074] Thompson, E.L. , Pitcher, L.E. , Niedernhofer, L.J. & Robbins, P.D. (2022) Targeting cellular senescence with Senotherapeutics: development of new approaches for skin care. Plastic and Reconstructive Surgery, 150(2 4S), 12S–19S. Available from: 10.1097/PRS.0000000000009668 36170431 PMC9529240

[joa70026-bib-0075] Thong, H.Y. , Jee, S.H. , Sun, C.C. & Boissy, R.E. (2003) The patterns of melanosome distribution in keratinocytes of human skin as one determining factor of skin colour. British Journal of Dermatology, 149(3), 498–505. Available from: 10.1046/j.1365-2133.2003.05473.x 14510981

[joa70026-bib-0076] Weinmüllner, R. , Zbiral, B. , Becirovic, A. , Stelzer, E.M. , Nagelreiter, F. , Schosserer, M. et al. (2020) Organotypic human skin culture models constructed with senescent fibroblasts show hallmarks of skin aging. Npj Aging and Mechanisms of Disease, 6(1). Available from: 10.1038/s41514-020-0042-x PMC7060247 32194977

[joa70026-bib-0077] Yamaguchi, Y. , Passeron, T. , Hoashi, T. , Watabe, H. , Rouzaud, F. , Yasumoto, K.I. et al. (2008) Dickkopf 1 (DKK1) regulates skin pigmentation and thickness by affecting Wnt/ β‐catenin signaling in keratinocytes. The FASEB Journal, 22(4), 1009–1020. Available from: 10.1096/fj.07-9475com 17984176

[joa70026-bib-0078] Yamaguchi, Y. , Takahashi, K. , Zmudzka, B.Z. , Kornhauser, A. , Miller, S.A. , Tadokoro, T. et al. (2006) Human skin responses to UV radiation: pigment in the upper epidermis protects against DNA damage in the lower epidermis and facilitates apoptosis. The FASEB Journal, 20(9), 1486–1488. Available from: 10.1096/fj.06-5725fje 16793869

[joa70026-bib-0079] Yamamoto, O. & Bhawan, J. (1994) Three modes of melanosome transfers in Caucasian facial skin: hypothesis based on an ultrastructural study. Pigment Cell Research, 7(3), 158–169. Available from: 10.1111/j.1600-0749.1994.tb00044.x 7971749

[joa70026-bib-0080] Yoshida, Y. , Hachiya, A. , Sriwiriyanont, P. , Ohuchi, A. , Kitahara, T. , Takema, Y. et al. (2007) Functional analysis of keratinocytes in skin color using a human skin substitute model composed of cells derived from different skin pigmentation types. The FASEB Journal, 21(11), 2829–2839. Available from: 10.1096/fj.06-6845com 17475923

